# Questionnaire survey of risk factors for recurrence of ocular inflammation in patients with uveitis after SARS-CoV-2 infection

**DOI:** 10.3389/fcimb.2023.1291991

**Published:** 2024-01-30

**Authors:** Zonghui Ma, Ying Chi, Chunying Guo, Jing Zhang, Liu Yang

**Affiliations:** Department of Ophthalmology, Peking University First Hospital, Beijing, China

**Keywords:** SARS-CoV-2 infection, uveitis, relapse, systemic immunosuppressive therapy, inflammatory activity

## Abstract

**Introduction:**

During the COVID-19 pandemic in China, the proportion of patients with uveitis who were infected with SARS-CoV-2 increased greatly. The impact of SARS-CoV-2 infection on patients with uveitis has not been fully described.

**Methods:**

A questionnaire on SARS-CoV-2 infection was sent to patients with uveitis to assess ocular and systemic conditions before and after infection. Chi-square analysis and multifactorial regression analysis were used to investigate the associations between each risk factor and the recurrence of uveitis after SARS-CoV-2 infection.

**Results:**

One hundred thirty-nine patients with noninfectious uveitis completed the questionnaire; 114 (82.0%) had COVID-19, and 27 (23.7%) had recurrent or exacerbated uveitis after COVID-19. There was a higher rate of recurrence or aggravation of ocular inflammation in patients who developed severe COVID-19 symptoms (severe group 8/20 vs. nonsevere group 19/94). There were significant differences in the rates of recurrence and aggravation between the two groups of patients who differed in terms of ocular inflammatory activity within 3 months prior to SARS-CoV-2 infection (χ2 = 10.701, *P*=0.001), as well as in the rates of recurrence and aggravation after cessation of systemic immunomodulatory therapy. After multifactorial regression analysis, patients with active ocular inflammation within 3 months prior to SARS-CoV-2 infection had a greater risk of recurrence or exacerbation of uveitis after COVID-19 (OR=4.298, *P*=0.002).

**Conclusion:**

The degree of ocular inflammatory activity within 3 months prior to SARS-CoV-2 infection may be a major factor influencing the recurrence or exacerbation of uveitis after infection. Interruption of medication should be minimized in patients with unstable inflammatory control.

## 1 Introduction

Coronavirus disease 2019 (COVID-19), which is caused by severe acute respiratory syndrome coronavirus 2 (SARS-CoV-2), emerged in December 2019 and quickly became a pandemic. SARS-CoV-2 infection affects mainly the respiratory system and can manifest as viral pneumonia or acute respiratory syndrome with multiple organ involvement(Guan et al., 2020; Huang et al., 2020). As the number of people infected with SARS-CoV-2 increases, there are more reports of ophthalmic manifestations in infected individuals(Gangaputra and Patel, 2020; Marinho et al., 2020; Xia et al., 2020; Zhang and Stewart, 2021).

Uveitis comprises a group of vision-threatening inflammatory diseases. It tends to recur due to various precipitating factors, and viral infection is one of its common triggers. After the peak of the COVID-19 epidemic, the number of outpatients with uveitis recurrence increased. There is insufficient evidence concerning whether SARS-CoV-2 infection affects the immune status of patients with uveitis and increases the chance of uveitis attack.

Some patients with uveitis need long-term immunomodulatory therapy to control the recurrence of inflammation or associated systemic disease. At present, the drugs of choice mainly include glucocorticoids, immunomodulators and biological agents. It has been suggested that glucocorticoid exposure is associated with a greater risk and serious consequences of COVID-19 in patients with uveitis; therefore, we may need to focus on limiting the use of immunomodulatory drugs during COVID-19 infection (Nørgård et al., 2021; Sun et al., 2022). Whether the discontinuation of immunomodulatory drugs affects the control of inflammation in patients with uveitis still needs further study.

The purpose of this study was to evaluate the systemic and ocular conditions of uveitis patients infected with COVID-19 through a questionnaire survey and to analyze the influence of various related factors on the stability of inflammation control. This study provides a reference for the management of uveitis patients with COVID-19, especially patients receiving immunomodulatory therapy.

## 2 Methods

This was a cross-sectional survey study involving a questionnaire survey of patients with uveitis followed up at the Peking University First Hospital Ophthalmology Department from January 15, 2020, to March 1, 2020. The period after December 2022, when the outbreak was concentrated in China, was chosen. This study was approved by the Ethics Committee of Peking University First Hospital. Participation in this study was voluntary. We informed patients of the purpose of this study before starting the formal study and obtained verbal consent to complete the questionnaire. If the patient in the course of the survey expressed unwillingness to continue, their participation was terminated.

This study was conducted using the group’s own questionnaire on SARS-CoV-2 infection. The questionnaire assessed the infection and ocular status of patients with uveitis during the SARS-CoV-2 epidemic. It consisted of five parts with 35 entries. The first section included demographic characteristics (5 items related to sex, age, usual address, weight and smoking history). The second section included information on the diagnosis of uveitis (8 items related to symptoms of uveitis onset, time of diagnosis, and type of uveitis). The third section included information on SARS-CoV-2 infection in patients with uveitis (11 items related to symptoms and treatment of SARS-CoV-2 infection). In particular, considering the large number of patients, the concentrated time of onset, and the relatively homogenous symptoms experienced during the COVID-19 epidemic in China, a substantial number of patients did not undergo nucleic acid or antigen testing to confirm the diagnosis. Patients in this group were diagnosed with SARS-CoV-2 infection based on their exposure history, time of onset, and symptoms. We regarded these patients as presumptive cases of COVID-19 infection. The fourth section concerned the recurrence of uveitis in patients with SARS-CoV-2 infection (5 entries related to the time of recurrence and medication). The fifth part of this study involved SARS-CoV-2 vaccination in patients with uveitis (6 entries related to the time of injection, adverse effects, and ocular manifestations). The questionnaires were distributed online through WeChat, QR code signs in clinics and telephone interviews. For specialized questions that could not be answered by or were inaccurately answered by the patients, we checked the patients’ clinical records for completeness and correctness. A total of 192 questionnaires were distributed, and 150 valid questionnaires were recovered; 11 patients with infectious uveitis were excluded, so a total of 139 valid questionnaires were obtained from patients with autoimmune-related uveitis.

All of the data were collated and analyzed using SPSS 25.0 statistical software. Descriptive analysis was used to perform a preliminary analysis of the data. To explore the risk factors for the recurrence or aggravation of uveitis after SARS-CoV-2 infection, we included 12 variable factors in our analysis model, namely, age, sex, smoking history, ocular manifestations, duration of uveitis, systemic disease, severity of SARS-CoV-2 infection (severe/nonsevere), ocular inflammatory activity (active/inactive) within three months prior to SARS-CoV-2 infection, medication use for systemic immunomodulatory therapy (glucocorticoids, immunosuppressants, and biological agents), and COVID-19 vaccination. We performed univariate analysis and binary logistic regression analysis for each variable. After excluding variables via the backward method (α=0.1), we found that ocular inflammation within three months prior to COVID-19 infection was significantly associated with the recurrence or exacerbation of uveitis. P values ≤ 0.05 indicated statistical significance.

## 3 Results

### 3.1 Demographic characteristics and diagnosis of uveitis

A total of 139 patients were included, with 53 (38.1%) males and 86 (61.8%) females. The demographic characteristics of the patients are summarized in **Table 1**. Information on the diagnosis of uveitis is summarized in **Table 2**. Anterior uveitis was the most common type of uveitis. The duration of disease was less than 5 years in 105 patients (75.5%), 5-10 years in 21 patients (15.1%) and more than 10 years in 13 patients (9.3%).

**Table 1:** Demographic data.

Gender (n, %)	
˙Male	53 (38.1%)
˙Female	86 (61.8%)
Age (n, %)	
˙no more than 18 years	25 (17.9%)
˙19 to 30 years	12 (8.6%)
˙31 to 40 years	30 (21.5%)
˙41 to 50 years	27 (19.4%)
˙51 to 60 years	23 (16.5%)
˙more than 60 years	22 (15.8%)
Permanent address(n, %)	
˙Beijing	94 (67.6%)
˙Domestic(except Beijing)	44 (31.7%)
˙Oversea	1 (0.7%)
Smoking history(n, %)	
˙History of smoking and still smoking	20 (14.3%)
˙History of smoking, quit smoking	10 (7.2%)
˙No history of smoking	109 (78.4%)

**Table 2:** Diagnostic information for uveitis.

Eyes with uveitis(n, %)	
˙Bilateral	50 (36.0%)
˙Unilateral	89 (64.0%)
Duration of follow up(n, %)	
˙less than 5 years	105 (75.5%)
˙5 to 10 years	21 (15.1%)
˙more than 10 years	13 (9.3%)
Uveitis Type (n, %)	
˙Anterior	66 (47.4%)
˙Intermediate	6 (4.3%)
˙Posterior	6 (4.3%)
˙Pan uveitis	43 (30.9%)
˙Unsure	18 (12.9%)
Underlying diagnosis (n, %)	
˙HLA-B27 related	20 (14.4%)
˙VKH	17 (12.2%)
˙TINU	6 (4.3%)
˙JIA	5 (3.6%)
˙Behcet	4 (2.9%)
˙Sarcoid	3 (2.2%)
˙Sjogrensyndrome	2 (1.4%)
˙Other*	7 (5.0%)
˙Idiopathic	75 (54.0%)

*Includes Fuch’s syndrome, systemic lupus erythematosus, rheumatoid and interstitial lung disease; VKH, Vogt-Koyanagi-Harada Syndrome; TINU, Tubulointerstitial Nephritis and Uveitis Syndrome; JIA, juvenile idiopathic arthritis.

Regarding the etiology of uveitis, the most common type was HLA-B27-associated uveitis (20 patients, 14.4%), followed by Vogt–Koyanagi–Harada disease (VKH) in 17 patients (12.2%), tubulointerstitial nephritis and uveitis syndrome (TINU) in 6 patients (4.3%), and uveitis associated with juvenile idiopathic arthritis (JIA) in 5 patients (3.6%). The other specific types included Fuch’s syndrome, systemic lupus erythematosus, and rheumatoid and interstitial lung disease (**Table 2**).

Symptoms of uveitis attack included eye redness (98 patients), eye pain (73 patients), photophobia (64 patients), decreased visual acuity (101 patients), light flashes (16 patients), eye floaters (34 patients), lacrimation (10 patients), blurred vision (2 patients), distorted vision (3 patients), eye swelling (3 patients), iridization (1 patient), eyelid edema (1 patient), and itchiness (1 patient); 2 patients had no obvious symptoms.

### 3.2 SARS-CoV-2 infection in patients with uveitis

Of the 139 patients, 114 (82.0%) reported that they had been infected with SARS-CoV-2. Infection occurred between November 2022 and January 2023 in the majority of patients (102, 89.5%). Among the patients who had been infected with SARS-CoV-2, 20 (14.3%) tested positive on the nucleic acid test, 55 (48.2%) tested positive on the antigen self-test, 20 (17.5%) had symptoms of COVID-19 without nucleic acid or antigen self-testing, and 19 (16.7%) presented with symptoms of COVID-19 without nucleic acid or antigen self-testing but with a positive nucleic acid or antigen self-test among family members. The 39 patients in the latter two groups were considered presumptive cases of COVID-19 infection. Twenty-five patients did not experience any symptoms during the epidemic, and their regular nucleic acid or antigen test results during this period were negative.

Among the patients who had been infected with SARS-CoV-2, only 2 (1.8%) were asymptomatic. Symptoms of infection included fever in 98 patients (86.0%), and the duration of fever was usually less than 3 days (91 patients). Systemic symptoms such as general pain (57.0%) and weakness (65.8%) and respiratory symptoms such as pharyngeal pain (59.6%) and cough (73.7%) were more common, while only a small number of patients had severe symptoms such as dyspnea (17.5%) and decreased blood oxygen (2.6%). Gastrointestinal symptoms such as diarrhea (14.0%) and vomiting (11.4%) were also not common (**Table 3**).

**Table 3:** Systemic symptoms during SARS-CoV-2 infection in patients with uveitis.

Symptom/Degree	None	Slight	Moderate	Serious	Severe
Generalized pain	49(43.0%)	24(21.1%)	23(20.2%)	16(14.0%)	2(1.8%)
Weakness	39(34.2%)	25(21.9%)	32(28.1%)	14(12.3%)	4(3.5%)
Pharyngeal pain	46(40.4%)	30(26.3%)	21(18.4%)	11(9.6%)	6(5.3%)
Cough	30(26.3%)	32(28.1%)	37(32.5%)	13(11.4%)	3(2.6%)
Dyspnea	94(82.5%)	15(13.2%)	5(4.4%)	0(0%)	0(0%)
Blood oxygen desaturation	111(97.4%)	2(1.8%)	1(0.9%)	0(0%)	0(0%)
Diarrhoea	98(86.0%)	9(7.9%)	3(2.6%)	4(3.5%)	0(0%)
Vomit	101(88.6%)	4(3.5%)	7(6.1%)	1(0.9%)	1(0.9%)
Loss of the sense of smell or taste	71(62.3%)	19(16.7%)	15(13.2%)	5(4.4%)	4(3.5%)

Three of the 20 patients with respiratory distress had a combined decrease in blood oxygen levels, and one of them had been hospitalized (not on a ventilator). A chi-square analysis of whether systemic medication was used to control uveitis was associated with severe symptoms that developed after SARS-CoV-2 infection, and the difference was not statistically significant (χ^2^ = 0.289, *P=0.591*).

### 3.3 Recurrence or aggravation of uveitis after SARS-CoV-2 infection and COVID-19 vaccination

Of the 114 patients infected with SARS-CoV-2, 27 (23.7%) experienced recurrence or aggravation of uveitis after SARS-CoV-2 infection. Of these patients, 20 had significant ocular symptoms, and 12 had active inflammation or an increased degree of inflammation according to eye examinations. The other 7 patients had no significant ocular symptoms but were revealed to have recurrence or aggravation of existing inflammation. There were 3 peaks of recurrence within 1 week (10 patients, 37.0%), approximately 1 month (8 patients, 29.6%) and approximately 2 months (4 patients, 14.8%) after infection.

Of the 94 patients who did not present with severe symptoms of COVID-19 during SARS-CoV-2 infection, 19 (20.2%) developed recurrence or exacerbation of uveitis after infection. Eight (40.0%) of the 20 patients who had severe symptoms experienced recurrence or aggravation of uveitis.

Thirty-eight patients had had an attack or persistent active inflammation within three months prior to COVID-19 infection. Nineteen (50.0%) patients had previous well-controlled inflammation and experienced an attack within three months prior to contracting COVID-19. Another 19 (50.0%) patients had persistent inflammation. Of these 38 patients, 16 (42.1%) experienced recurrence or aggravation of uveitis after SARS-CoV-2 infection. However, of the 76 patients with inactive ocular inflammation within three months prior to COVID-19 infection, 11 (14.5%) experienced recurrence or aggravation.

There were significant differences in the rates of recurrence and aggravation between patients who had active inflammation and those who did not within three months prior to infection (χ2 = 10.701, *P*=0.001).

A total of 46 patients were treated with systemic drugs for ocular inflammation at the time of SARS-CoV-2 infection, including 32 patients treated with glucocorticoids, 30 patients treated with immunomodulators, and 21 patients treated with biological agents. Among the patients who received systemic medications, glucocorticoids alone were used in 7 patients, immunomodulators alone were used in 5 patients, and adalimumab alone was used in 6 patients. Thirteen patients were treated with glucocorticoids combined with immunomodulators, 3 patients were treated with glucocorticoids combined with biological agents, 3 patients were treated with immunomodulators combined with biological agents, and 9 patients were treated with all three types of agents. Of these 46 patients treated with systemic drugs for ocular inflammation control, 19 discontinued the drug during the period of SARS-CoV-2 infection. We performed Pearson’s chi-square analysis to compare postinfection uveitis activity between two groups of patients treated with or without systemic immunomodulatory drugs during COVID-19 infection. No significant difference was found (χ2 = 0.246, *P*=0.620).

We further focused on the relationships between ocular inflammatory activity prior to SARS-CoV-2 infection and between medication use and recurrence (or aggravation) after infection. We discovered that, of the 38 patients with inflammatory activity prior to SARS-CoV-2 infection, 17 were using systemic agents at the early stage of infection, and 9 patients discontinued the drug. Five of those who discontinued treatment experienced recurrence or aggravation of uveitis after SARS-CoV-2 infection. In contrast, of the 29 patients on systemic drugs who were free of inflammation within 3 months prior to infection, 10 patients discontinued the drug, but none had uveitis relapse (*P*=0.011).

Of the 139 patients, 107 (77.0%) were vaccinated against COVID-19. Thirteen patients (12.1%) developed a recurrence of uveitis within 2 weeks of vaccination, and 3 of these 13 patients also developed a recurrence of uveitis after SARS-CoV-2 infection (**Table 4**).

**Table 4:** Data of COVID-19 vaccination.

Vaccination times(n, %)	
˙Zero	32 (23.0%)
˙One	1 (0.7%)
˙Two	38 (27.3%)
˙Three	61 (43.8%)
˙Four	7 (5.0%)
Any recurrence of uveitis within 2 weeks of vaccination(n, %)	13 (12.1%)

We performed unifactorial and multifactorial analyses of factors that could be associated with the recurrence or exacerbation of uveitis. In multifactorial logistic stepwise regression, ocular inflammatory activity (active/inactive) within three months prior to SARS-CoV-2 infection was retained (OR=4.298, *P*=0.002), and the remaining factors were excluded (**Table 5**).

**Table 5:** Analysis of factors associated with recurrence or exacerbation of uveitis.

Variables (reference value)		Univariate analysis		Multifactor regression analysis	
		OR (95%)	*P*	OR (95%)	*P*
**Gender** (Female)		2.245(0.822-6.130)	0.114		
**Age**		1.013(0.988-1.038)	0.310		
**Smoking history** (No history of smoking)	Quit smoking	0.507(0.058-4.437)	0.540		
	Still smoking	0.830(0.213-3.236)	0.788		
**Eyes with uveitis** (Bilateral)		1.107(0.445-2.758)	0.827		
**Duration of uveitis** (less than 5 years)	5 to 10 years	1.017(0.328-3.151)	0.977		
	Over 10 years	0.555(0.113-2.717)	0.467		
**Systemic disease**		1.312(0.519-3.320)	0.566		
**Severity of COVID-19 infection** (non-severe)		2.632(0.943-7.345)	0.065		
**Ocular inflammatory activity before SARS-CoV-2 infection**		4.298(1.735-10.647)	0.002	4.298(1.735-10.647)	0.002
**Use of glucocorticoids**		1.105(0.427-2.859)	0.836		
**Use of immunosuppressants**		1.571(0.615-4.-18)	0.345		
**Use of biological agents**		0.716(0.219-2.346)	0.581		
**Vaccination times** (Zero)	One	/	/		
	Two	0.641(0.171-2.405)	0.510		
	Three	1.351(0.453-4.027)	0.589		
	Four	0.833(0.078-8.947)	0.880		

## 4 Discussion

A questionnaire survey was conducted to investigate the systemic symptoms and factors associated with the recurrence of ocular inflammation in patients with uveitis after COVID-19 infection. We found that unstable uveitis before SARS-CoV-2 infection may be associated with an increased risk of uveitis recurrence or aggravation.

In this study, patients who received systemic immunomodulatory therapy when COVID-19 infection occurred were compared with patients who did not receive systemic medication. Moreover, there was no significant difference between the two groups in terms of the risk of severe symptoms of COVID-19. Moreover, there was no difference when comparing the recurrence or aggravation of uveitis after COVID-19 infection. Studies have shown that uveitis patients receiving systemic use of glucocorticoids, especially those receiving higher doses or long-term use, may face a greater risk of COVID-19 and serious outcomes(Nørgård et al., 2021; Sun et al., 2022). However, long-term treatment with TNF-α and IL-6 inhibitors or other biological agents may have a protective effect on the severity of COVID-19 disease(GianFrancesco et al., 2020; Santos et al., 2021). Most of the uveitis patients who received systemic immunomodulatory therapy in this study were treated with glucocorticoids combined with immunomodulators and/or biological agents. Therefore, the effect of a single drug could not be differentiated. Population-based studies from Denmark and Sweden have shown an increased risk of hospitalization for COVID-19 in patients with immune-mediated inflammatory diseases compared with the general population (Bower et al., 2021; Cordtz et al., 2021). However, a US study found that uveitis itself was not associated with COVID-19 infection or severity after adjusting for demographic variables, comorbidities, and medications (Miller et al., 2022). In other words, the specificity of an individual’s immunosuppression status, rather than the presence of autoimmune disease, is associated with the risk of COVID-19 infection and poor outcomes. Therefore, the heterogeneity of the results of each study may be due to the individual differences in uveitis patients, the medication used, and the degree of inflammation control.

Patients were divided into two groups according to ocular inflammatory activity before COVID-19 infection: those without active inflammation and those with an attack or persistent active inflammation within three months prior to COVID-19 infection. We found that patients who experienced an attack or persistent active inflammation not long before contracting COVID-19 were more likely to experience ocular inflammation relapse or exacerbation after COVID-19. This was expected, as our definitions of recurrent, chronic and remission uveitis are generally limited to 3 months (Jabs et al., 2005). Compared with patients without active inflammation, patients with recurrent or persistent active inflammation within three months prior to COVID-19 are still in a state of inflammation or are in a state of instability. At this time, viral infection or a systemic inflammatory response is more likely to induce or aggravate ocular inflammation.

In addition, when comparing the inflammation recurrence rate between patients who discontinued drugs and patients who did not during COVID-19, there were differences between the two groups. However, the number of patients was not large enough to obtain statistically significant results. In the group with active ocular inflammation within three months prior to COVID-19, nine patients discontinued their medication during the infection, and five of them experienced a recurrence or increase in inflammation after the infection. Among the 29 patients who did not have active inflammation, none of the 10 patients who discontinued the drug experienced relapse. In the study by Aniruddha et al., 25 of 121 patients with active inflammation who were taking immunomodulatory agents experienced a relapse of inflammation because of dose reduction or withdrawal during COVID-19 infection (Agarwal et al., 2022). Patients in the unstable phase of inflammation are more dependent on drugs, and drug withdrawal itself or exposure factors (such as infection) after drug withdrawal may be more likely to have an impact on patients. Definitive conclusions should be supported by studies with larger samples.

According to the definition of severe symptoms in the literature, patients who experienced dyspnea and/or decreased blood oxygenation during the course of COVID-19 infection were defined as those with severe COVID-19 infection, whereas patients who did not have either of these manifestations were defined as those with nonsevere COVID-19 infection (Lamontagne et al., 2020; Hu et al., 2022). A comparison of the ocular data of the two groups after infection revealed that there was a greater rate of recurrence or aggravation of ocular inflammation in patients who developed severe COVID-19 symptoms. However, we conducted a multifactorial regression analysis to exclude other factors from interference, and these factors had no effect on the recurrence of ocular inflammation. Previous studies have shown that the severity of COVID-19 is closely related to cytokine storms. The levels of IL-6, IL-10, TNF-α, IL-1β, IL-4, IL-8, IL-17 and other cytokines are significantly greater in severe patients than in mild patients (Hu et al., 2022). In particular, the serum level of IL-6 is positively correlated with the severity of COVID-19 and is considered to be an indicator of disease severity (Henry et al., 2020; Ghofrani Nezhad et al., 2023). However, Th 17 cells and their related cytokines, such as IL-6, IL-10, IL-17 and TNF-α, are important inflammatory mediators in autoimmune diseases, including uveitis, and are thought to drive intraocular inflammation (Luger et al., 2008; Weinstein and Pepple, 2018). Elevated levels of IL-17 have been detected in the eyes of patients with VKH, HLA-B27 and Behcet uveitis, as well as in the serum of patients with other immune-mediated uveitis. IL-6 and TNF-α are attractive potential therapeutic targets for ocular inflammatory diseases (El-Asrar et al., 2011; Jawad et al., 2013). Therefore, the increased levels of many cytokines in patients with severe COVID-19 may also induce inflammation in patients with uveitis, leading to the recurrence or aggravation of ocular inflammation after infection. However, there are many influencing factors that still need to be clarified by further research.

Since the widespread introduction of the COVID-19 vaccine, there has been ongoing discussion about whether COVID-19 vaccination will increase the risk of uveitis. There have been case reports of VKH following vaccination (Bolletta et al., 2021; Saraceno et al., 2021; Alhamazani et al., 2022), as well as cases of vaccine-associated recurrent uveitis (Jain and Kalamkar, 2021). However, a retrospective study among 120 patients showed that inactivated COVID-19 vaccination did not increase the risk for exacerbations in patients with uveitis, regardless of systemic immunosuppressive therapy (Song et al., 2022). However, it has not been reported whether COVID-19 vaccination has an impact on the risk of uveitis recurrence after COVID-19 infection. In this study, there was no significant difference in the recurrence rate of uveitis between vaccinated and unvaccinated patients. However, as a retrospective questionnaire survey, there was insufficient evidence of a relationship between them.

This study has the following limitations. First, this study included 150 patients with uveitis who were registered and followed up regularly at Peking University First Hospital. The sample size was limited, and our patients were mainly from northern China, mainly from Beijing; therefore, the sample data may not represent uveitis patients nationwide. Second, this study was conducted after the peak of the SARS-CoV-2 epidemic in China. During the pandemic, only a small number of patients were diagnosed by positive nucleic acid tests, while nearly half of the patients were diagnosed by antigen self-tests; these tests are slightly less accurate but more convenient and rapid. For patients who did not have SARS-CoV-2 symptoms, some could only judge that they had no symptoms of infection but lacked reliable nucleic acid or self-test antigen results. Therefore, there may have been potential classification errors in terms of whether patients were infected with SARS-CoV-2. Third, in terms of the data on symptoms and severity of COVID-19 infection, although we provided detailed symptoms and severity grading, the results still depended on the subjective feelings of patients. Therefore, the severity of symptoms may have differed from the objective situation due to differences in baseline feelings and tolerance levels.

In conclusion, COVID-19 may increase the risk of recurrence of uveitis. For patients with unstable inflammation control, we should be more cautious about the recurrence or aggravation of inflammation caused by COVID-19. There is insufficient evidence to confirm that severe infections and immunomodulatory therapy affect the recurrence of uveitis.

## Data availability statement

The raw data supporting the conclusions of this article will bemade available by the authors, without undue reservation.

## Ethics statement

The studies involving humans were approved by Biomedical Research Ethics Committee/Peking University First Hospital. The studies were conducted in accordance with the local legislation and institutional requirements. Written informed consent for participation in this study was provided by the participants and/or their legal guardians/next of kin.

## Author contributions

ZM: Data curation, Formal analysis, Investigation, Methodology, Writing – original draft, Writing – review & editing. YC: Data curation, Funding acquisition, Methodology, Supervision, Writing – original draft, Writing – review & editing. CG: Data curation, Formal analysis, Investigation, Methodology, Writing – review & editing. JZ: Data curation, Formal analysis, Investigation, Methodology, Writing – review & editing. LY: Data curation, Project administration, Supervision, Writing – review & editing.
